# Fast and robust 3D T1 mapping using spiral gradient shape and continuous radio-frequency excitation at 7 T : Application on cardiac Manganese Enhanced (MEMRI) MRI in mice

**DOI:** 10.1186/1532-429X-17-S1-P258

**Published:** 2015-02-03

**Authors:** Charles Castets, Emeline Julie Ribot, William Lefrançois, Aurélien J Trotier, Jean-Michel Franconi, Sylvain Miraux

**Affiliations:** UMR5536, CNRS, Bordeaux, France

## Background

Mapping the longitudinal relaxation time (T1) is a promising quantitative tool for the detection of myocardial pathologies either in mouse or human. T1 maps are usually obtained in 2D in order to reduce the total scan time. However, to diagnose myocardial infarction it might be useful to obtain the T1 maps in three dimensions (3D) which require a long acquisition time. In this project a new 3D T1 mapping method based on Look-Locker protocol using continuous radio frequency (RF) excitation and spiral gradient shape has been developed. This method allowed to obtain 3D maps with high spatial (200 x 200 x 300 μm^3^) & temporal resolution (<12 min). Moreover, manganese injections demonstrated the capacity of this method to detect T1 changes in the mouse heart.

## Methods

All the mice were imaged on a 7T horizontal magnet (Bruker Biospin). The acquisition sequence begin with an inversion pulse allowing to inverse the longitudinal magnetization. Then, a continuous RF pulse train synchronized with the mouse electrocardiogram was applied to acquire signals at different inversion times. To reduce the acquisition time, two spiral interleaves were acquired per RR interval. For each experiment twenty-three 3D images were acquired during the signal relaxation. A new fitting protocol has also been developed which takes into account the lower magnetization equilibrium induced by the continuous RF excitation. The precision of this protocol was validated on a phantom with various concentrations of MnCl2. T1 were measured using a gold standard inversion-recovery (IR) and the new developed method. To test the robustness of this new method in vivo, T1 maps were acquired after successive injections (6) of 10μL of MnCl2 at 50mM in 5 mice. The following imaging parameters were used : Matrix=96x96x48 ; field-of-view=20x20x15mm^3^ ; TR/TE=6/1.5ms; Bandwidth=300kHz; Flip-angle=10°.

## Results

**Figure 1:**
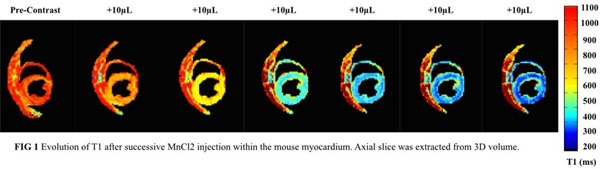
Figure 1

In vitro results showed a good correlation between the T1 measured with IR and the new method. The R1 of MnCl2 was equal to 6.37mM.s-1 for IR and 6.40mM.s-1 for the new method. 3D T1 maps were obtained for 5 mice. An average value of 1100±70 ms for the myocardium was measured without injection of contrast agent. The T1 decrease was quantified during successive injections of MnCl2 allowing to follow the manganese uptake in 3D (FIG 1).

## Conclusions

The developed method allowed to obtain 3D T1 maps in less than 12 minutes. In vivo experiments demonstrated the efficiency of this protocol to quantify Mn concentration in the whole mouse heart. This technique will be validated on pathological models.

## Funding

This study was supported by LABEX TRAIL.

